# Serial assessment of early antibody binding to decellularized valved allografts

**DOI:** 10.3389/fcvm.2022.895943

**Published:** 2022-08-09

**Authors:** Firdavs Oripov, Robert Ramm, Christine Falk, Tobias Goecke, Johannes Ebken, Ramadan Jashari, Dietmar Böthig, Alexander Horke, Murat Avsar, Dmitry Bobylev, Axel Haverich, Andres Hilfiker, Samir Sarikouch

**Affiliations:** ^1^Leibniz Research Laboratories for Biotechnology and Artificial Organs (LEBAO), Hannover Medical School, Hanover, Germany; ^2^Institute of Transplant Immunology, Hannover Medical School, Hanover, Germany; ^3^Department of Cardiothoracic, Transplantation and Vascular Surgery, Hannover Medical School, Hanover, Germany; ^4^European Homograft Bank, Clinique Saint-Jean, Brussels, Belgium

**Keywords:** heart valve replacement, homograft, decellularization, immune system, antibodies

## Abstract

**Objectives:**

Decellularized homograft valves (DHV) appear to elicit an immune response despite efficient donor cell removal.

**Materials and methods:**

A semiquantitative Dot-Blot analysis for preformed and new recipient antibodies was carried out in 20 patients following DHV implantation on days 0, 1, 7, and 28 using secondary antihuman antibodies. Immune reactions were tested against the implanted DHV as well as against the stored samples of 5 non-implanted decellularized aortic (DAH) and 6 pulmonary homografts (DPH).

**Results:**

In this study, 20 patients (3 female and 17 male patients) were prospectively included, with a median age of 18 years and an IQR of 12–30 years. Six patients received DPH and 14 received DAH. The amount of antibody binding, averaged for all patients, decreased on post-operative days 1 and 7 compared to pre-operative values; and on day 28, antibody binding reached close to pre-operative levels (16.8 ± 2.5 on day 0, 3.7 ± 1.9 on day 1, 2.3 ± 2.7 on day 7, and 13.2 ± 3.7 on day 28). In comparison with the results in healthy controls, there was a higher amount of antibody binding to DAH than to DPH. The mean number of arbitrary units was 18.4 ± 3.1 in aortic and 12.9 ± 4.5 in pulmonary DHV (*p* = 0.140). Male patients exhibited higher antibody binding to aortic DHV than female patients (19.5 ± 2.1 vs. 1.6 ± 6.7). The *p*-value calculation was limited, as only two female patients received DAH. There was no correlation between the amount of overall antibody binding to DHV with respect to donor age (Kruskal–Wallis test *p* = 0.550). DHV recipients with a sex mismatch to the donor showed significantly less antibody binding (6.5 ± 1.8 vs. 13.7 ± 1.8; *p* = 0.003). Our main finding was an increase in antibody binding in younger patients receiving decellularized aortic allografts. This increase was higher in patients with early degeneration signs but was not specific to the individual DHV implanted nor previous DHV implantation. Antibody binding toward explanted DHV was significantly increased in implicating antibody-mediated DHV degeneration.

**Conclusion:**

Serial assessment of tissue-specific antibody binding revealed an increase in some patients within 4 weeks after surgery, who subsequently developed early signs of allograft degeneration. Further studies with larger sample sizes are needed to confirm the prognostic relevance of increased antibody activity in addition to targeted research efforts to identify the molecular agents triggering this type of antibody response.

## Introduction

Decellularized homografts are currently the only clinically applied tissue-engineered heart valves and have shown superior performance in comparison with conventional cryopreserved allografts in a recent meta-analysis for pulmonary valve replacement. Reoperation rates in 1,418 patients who underwent outflow tract reconstructions with decellularized heart valves (DHV) were significantly lower than in the 2,725 patients who received conventional allografts (4.8 vs. 7.4%; pooled risk ratio (RR) 0.55, 95% CI: 0.36 to 0.84; *p* = 0.0057) (1).

Despite almost complete decellularization, documented by standard microscopy and very low residual DNA content, DHV seem to elicit an immune response that appears to be stronger in younger recipients, indicated by progressing valve dysfunction (2). This has been observed especially in decellularized aortic homografts (DAH) recipients and may be explained by the fact that aortic homografts are considerably thicker, thereby potentially carrying more antigenic material. Classic T-cell-mediated immune response, considered to be the leading mechanism for the usually relatively slow mode of degeneration in cryopreserved heart valves, may not be the key mechanism in DHV degeneration.

In an initial analysis of immune reactions to DHV in children and young adults, we did not find significant changes in peripheral cell counts, which were followed up to 3 years after implantation, for mature T-(CD3 +), B-(CD19 +), and natural killer-(CD16 + /CD56 +) cells and for T helper-(CD4 +) and cytotoxic T-cell-(CD8 +) subsets (3). In a systematic *in vivo* recellularization analysis of explanted DHV and biopsy material, we did not observe classic T-cell infiltration, even in the case of an adult patient following a Ross procedure with rapid degeneration of his pulmonary DHV (4). This led to the hypothesis that DHV degeneration may be predominantly antibody-mediated, based on the observed temporal increase in DHV-specific IgG levels in 14 patients following decellularized DHV (5) and the results of a newly developed Dot-Blot technique for decellularized porcine heart valves (6). These results indicated substantial binding of preformed human antibodies to decellularized xenogeneic heart valves with considerable variance in the individual immune response toward specific porcine samples.

In our initial analysis of preformed antibody binding to DHV in healthy controls with no known contact with allogeneic tissue, we observed that DHV bind preformed recipient antibodies, which might elicit an immune response. There was considerable interindividual heterogeneity and also wide variation between specific individual decellularized human specimens. Age played an important role, as expected, with a higher immune response in younger individuals (7). Significantly higher binding of preformed antibodies was observed in male subjects. These findings correlated with a recent study from Netherlands on the outcome of standard cryopreserved pulmonary homografts, which described considerably poorer outcomes in male patients (8).

The purpose of the current analysis was to measure antibody binding to DHV in patients with repaired congenital heart disease. Our aim was to (1) compare the amount of baseline preformed antibody binding in patients who had received different patch material and different heart valve substitutes to levels of antibody binding in healthy controls, and (2) to analyze the early impact of DHV implantation in these patients using the Dot-Blot test and echocardiography.

## Materials and methods

### Study design and patient selection

The study was registered with and approved by the local ethics committee (EC No. 7871_BO_S_2018). Patients were not selected randomly, but 20 consecutively operated patients receiving a decellularized homograft in the period October 2019 to May 2020 were included following informed consent provided by the patients or the parents. Since 2008, there have been a total of 199 implantations of decellularized aortic homografts and 236 implantations of decellularized pulmonary homografts at Hannover Medical School.

A serial assessment of the individual *in vivo* immune response toward decellularized homograft valves (DHV) was performed using a Dot-Blot technique at 4 time points: pre-operation, post-operation on day 1, before discharge at day 7, and at day 28. Five patients opted against attending the last session of the blood withdrawal due to the potential risk of COVID-19 infection in the pandemic situation of autumn 2020.

Individual antibody binding for each patient was measured at the above outlined time points using stored samples of 5 non-implanted decellularized aortic (A1–A5) and 6 non-implanted pulmonary homografts (P1–P6). In 18 patients, it was possible to acquire tissue samples from the implanted DHV at the time of the operation, allowing these samples to be included in the serial assessments. In four patients who underwent DHV exchange or explantation, the assessment also included samples from the explanted allograft. In one of these patients, a retained DAH sample prior to implantation was also available.

Patient characteristics are displayed in Table 1. Patient No. 20 underwent a bilateral lung transplantation 18 months prior to a DAH procedure and was therefore on an immunosuppressive medication regimen. None of the other included patients were receiving immunosuppressive medication, e.g., steroids, at the time of operation and 6 months prior to the surgery. In addition, none of the patients suffered from metabolic syndrome, diabetes mellitus, or acquired heart disease, such as systemic or pulmonary hypertension or coronary heart disease.

**TABLE 1:** Patient cohort characteristics.

Patient no.	Sex	Age (years)	Valve	Indication	Blood group	Follow-up
1	M	15	Aortic	Stenosis, first AVR	O +	15 months, DAH echogenicity↑
2	M	11	Aortic	Stenosis, first AVR	A −	15 months, DAH intact
3	M	16	Pulmonary	Regurgitation, redo DPH	A +	13 months, DPH echogenicity↑
4	M	14	Pulmonary	Regurgitation, first PVR	A +	10 months, DPH intact
5	M	11	Aortic	Stenosis, first AVR	A +	18 months, DAH intact
6	M	9	Aortic	Regurgitation, first AVR	O +	14 months, DAH echogenicity↑
7	M	6	Aortic	Stenosis, redo DAH	O −	23 months, DAH intact
8	F	17	Pulmonary	Regurgitation, first PVR	B +	17 months, DPH intact
9	M	16	Aortic	Stenosis, first AVR	O +	18 months, DAH intact
10	M	15	Aortic	Stenosis, redo DAH	O +	13 months, DAH echogenicity↑
11	M	20	Pulmonary	Regurgitation, redo DPH	A +	17 months, DPH intact
12	M	19	Pulmonary	Regurgitation, first PVR	A +	20 months, DPH intact
13	F	21	Aortic	Stenosis, first AVR	O +	24 months, DAH intact
14	M	46	Aortic	Regurgitation, first AVR	O +	16 months, DAH intact
15	M	30	Pulmonary	Stenosis, fourth PVR	O +	13 months, DPH intact
16	M	20	Aortic	Stenosis, first AVR	O −	25 months, DAH intact
17	M	30	Aortic	Stenosis, first AVR	O +	12 months, DAH intact
18**	M	44	Aortic	Stenosis, first AVR	O −	12 months, DAH intact
19	M	41	Aortic	Regurgitation, first AVR	O +	20 months, DAH intact
20*	F	29	Aortic	Regurgitation, first AVR	A +	15 months, DAH intact

Pat *20 underwent bilateral lung transplantation 18 months prior to DAH, Pat **18 underwent chemotherapy 6 months prior to DAH.

DAH, decellularized aortic homograft; DPH, decellularized pulmonary homograft; AVR, aortic valve replacement; PVR, pulmonary valve replacement.

All 20 patients underwent echocardiographic follow-up over a mean period of 16.0 ± 4.7 months following DHV implantation.

### Decellularized homografts

The decellularized homografts used in this study were processed by corlife oHG,^1^ a Hannover-based tissue establishment. The decellularization process comprised approximately 30 different steps using a detergent-based, non-cryopreservation approach as described previously (9). Pulmonary allografts were treated under shaking conditions with a solution of 0.5% sodium deoxycholate (Sigma) and 0.5% sodium dodecyl sulfate (Carl Roth) for 36 h at room temperature. Homografts were washed with 0.9% NaCl solution and stored at 4°C until implantation. Histologically, each homograft was assessed after processing, and the residual dsDNA content was measured before and after processing prior to final release, the upper limit for release being 25 ng/ml. Reference samples of all homografts were stored for at least 1 year according to German law.

The non-implanted homografts used in this study were procured by the European Homograft Bank (EHB), Clinic St. Jean, Brussels (Material Transfer Agreement 180718); the implanted homografts were procured by the EHB and the Deutsche Gesellschaft für Gewebetransplantation (DGFG Hannover).

### Dot-Blot technique

The DHV tissues (approximately 500 mg pulverized tissue) were solubilized using collagenase II digestion, dotted on nitrocellulose membranes, and exposed to human serum. Bound human serum antibodies were detected using antihuman secondary antibodies, enabling detection with an ECL reagent. The assay was conducted as previously published with minor modifications (7).

Decellularized homograft valvess were dotted in triplicates (10 μg/dot in 200 μl TBS), and a dilution series of human serum (1:16.000 to 1:512.000) serving as the standard was placed in duplicates on each membrane. Loaded glutaraldehyde (Polysciences Inc., Warrington, FL, United States) fixed and blocked membranes were incubated in human serum (1:400), diluted in 10% non-fat dry milk/TBS at 4°C overnight. Bound antibodies were detected by applying goat antihuman IgG, IgA, IgM-HRP secondary antibody diluted at 1:50,000 and ECL substrate (PerkinElmer, Waltham, MA, United States). Chemiluminescence signals were imaged with the ChemiDoc imager (Bio-Rad, Hercules, CA, United States; applied software settings: “chemi Blot,” 26 photos, 56 s exposure time, accumulation signal mode) and densitometrically quantified (in arbitrary units) with Image Lab 5.0 software (Bio-Rad, Hercules, CA, United States).

### Test reproducibility and statistics

Technical triplicates were generated for all Dot-Blots in this study. The experimental variance was assessed using Cronbach’s Alpha as an estimate for Tau-equivalent reliability. Cronbach’s Alpha is a function of the number of items in a test, the average covariance between item pairs, and the variance of the total score. A reliability score greater than 0.8 indicates good internal consistency; a Cronbach’s Alpha >0.9 indicates excellent consistency.

The test robustness and reproducibility of the established Dot-Blot method have been extensively tested in decellularized xenogeneic heart valves (6). The resulting interassay variance of 9.5% and the intra-assay variance of 9.2% showed that the test is highly reproducible. As the binding of the secondary antihuman antibodies after decellularization can lead to false-positive results due to remaining donor antibodies in the DHV, we introduced a baseline by loading a membrane with DHV that was not exposed to patient serum and subtracted the result for this membrane from each specific serum-incubated specimen. The negative values after baseline subtraction observed in some cases can be explained by steric overlays between remaining donor antibodies and serum antibodies.

We included all medians of the arbitrary antibody-binding units that resulted from matching triplicates of sera of the 20 DHV recipients at the 4 standardized time points with 11 samples of DHV non-implanted test valves and 18 samples of the implanted valves. In two patients, it was not possible to extract material from the implanted valve without causing valve function impairment.

SPSS 27 (IBM Corporation, Somer, NY, United States), MedCalc (Statistical Software version 20.015 from MedCalc Software bv, Ostend, Belgium; 2020)^2^ and GraphPad Prism 5.0a (GraphPad Software, San Diego, CA, United States) were used for statistical analyses. Summaries of numeric data are given as means and standard errors of the mean or median and interquartile range; we used 2-sided Student’s *t*-tests and the Mann–Whitney or Kruskal–Wallis tests (as appropriate) for univariate comparisons and binary logistic regression to analyze statistical significances and weights of factors, respectively. Differences were considered to be statistically significant for values of *p* ≤ 0.05.

## Results

In the current study, 20 patients, with a median age of 18 years and an interquartile range of 12–30 years, were prospectively included. The group was not balanced with respect to sex and included 3 female and 17 male patients. Six patients received DPH and 14 received DAH. Fifteen patients (75%) had undergone at least one previous surgical procedure, and one patient had received bilateral lung transplantation. Four patients (20%) had already obtained a decellularized allograft in the past. Five patients did not return for the blood sampling on day 28 due to the potential risk of infection during the third wave of the 2020 COVID-19 pandemic. The study cohort characteristics are described in Table 1.

### Comparison of overall antibody binding to healthy controls

The amount of preformed antibodies which bound to DHVs preoperatively were higher in males than in the sera of female patients. The mean number of arbitrary units was 7.6 ± 4.7 (SEM, standard error of mean) in female patients and 18.5 ± 2.9 in male patients (*p* = 0.060).

The low number of female patients (*n* = 3) limited the comparison to healthy controls. The data for healthy controls were taken from our previous publication (7). However, pre-operative antibody binding in male patients was almost identical to healthy male controls (18.0 ± 3.0), whereas female controls had higher antibody binding (13.6 ± 3.2).

In addition to the results in healthy controls, there was a higher amount of antibody binding to aortic DHVs compared with pulmonary DHVs in the study cohort. The mean number of arbitrary units was 18.4 ± 3.1 in aortic DHV and 12.9 ± 4.5 in pulmonary DHV. This did not reach statistical significance (*p* = 0.140), and a robust comparison was hindered by the low number of implanted pulmonary DHV (*n* = 6).

Male patients exhibited higher antibody binding to aortic DHV than female patients (19.5 ± 2.1 vs. 1.6 ± 6.7). The *p*-value calculation was, however, limited by the fact that only two female patients received DAH, a ratio in line with the threefold higher incidence of aortic valve disease in male patients.

**FIGURE 1:**
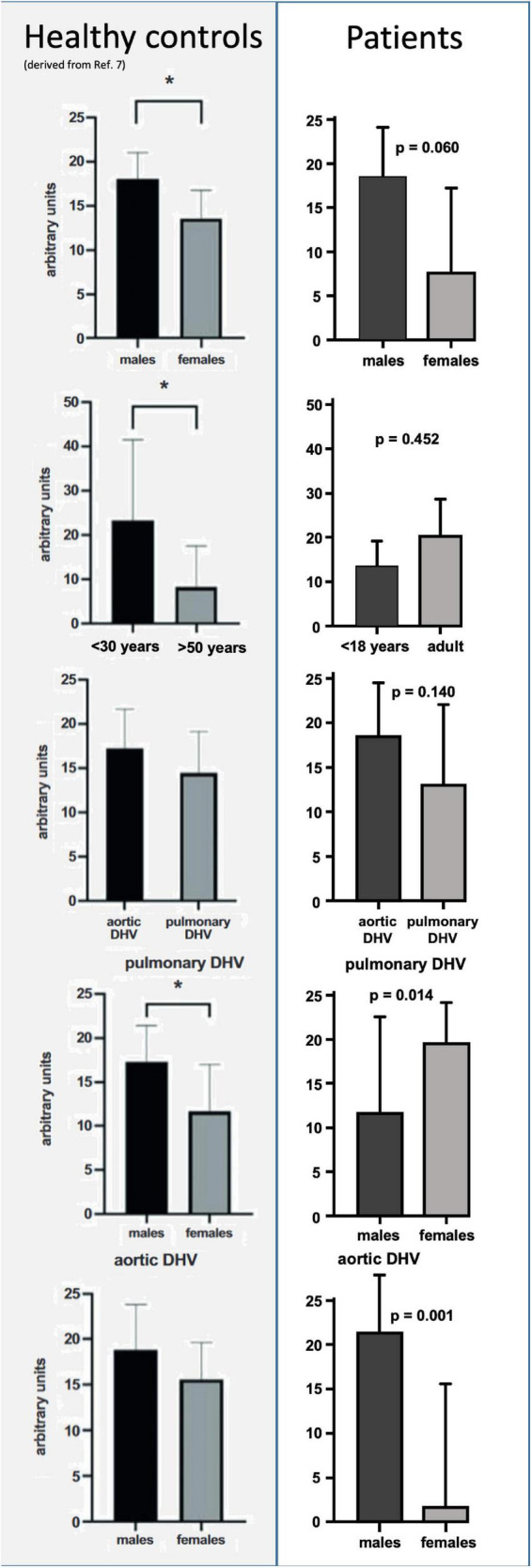
Comparison of preformed antibody binding in minced decellularized allograft samples between healthy controls and patients who had received decellularized allografts according to sex, age, and valve position. Data from healthy controls were taken from our previous publication (7). Please note the different age categories in healthy controls, which were all over 18 years of age. Asterisks indicate statistical significance.

Figure 1 provides an overview of generally unspecified antibody binding pre-operatively and in comparison with the results in healthy controls. Please note the different age categories in the control group, which included participants over 18 years of age.

### Correlation of baseline antibody binding according to donor age and recipient blood group

There was no correlation between the amount of overall antibody binding with respect to donor age, calculated using the Kruskal–Wallis test, with a *p-value* = 0.550.

**FIGURE 2:**
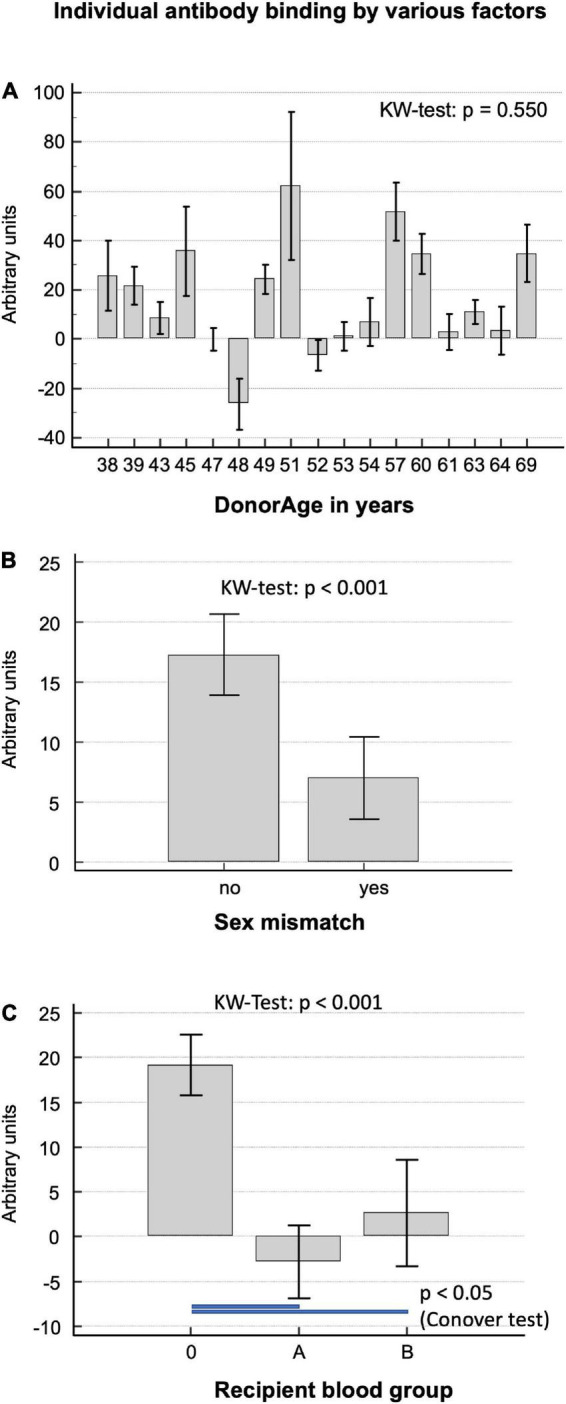
**(A)** Correlation of baseline antibody binding in patients who had received decellularized allografts according to donor age. **(B)** Antibody binding in patients with and without a sex mismatch to the DHV donor. **(C)** Antibody binding in patients differentiated by recipient blood group.

Figure 2A shows the high level of variance in antibody binding between the 18 implanted decellularized allografts and the respective donor age. Interestingly, DHV recipients with sex mismatch to the donor showed significantly less antibody binding (6.5 ± 1.8 vs. 13.7 ± 1.8; *p* = 0.003), as seen in Figure 2B.

Decellularized homograft valves recipients with blood group O showed significantly more antibody binding (median; IQR 11.6; −5.6 to 35.5) than A (*p* < 0.001; −7.7; −25.2 to 9.3) and B (*p* < 0.031; 9.1; −15.5 to 18.3), as seen in Figure 2C.

### Development of antibody-binding amounts within the first month after decellularized homograft valves implantation in correlation to age, valve position, and echocardiographic follow-up

The amount of antibody binding, averaged for all patients, decreased on post-operative days 1 and 7 compared with pre-operative values and returned to levels close to pre-operative values on post-operative day 28: 16.8 ± 2.5 on day 0, 3.7 ± 1.9 on day 1, 2.3 ± 2.7 on day 7, and 13.2 ± 3.7 on day 28. DHV recipients with previous procedures showed less antibody binding in general (no previous operation 15.9 ± 36.5, 1 previous operation 7.9 ± 44.2, and 2 previous operations 9.2 ± 21.6).

**FIGURE 3:**
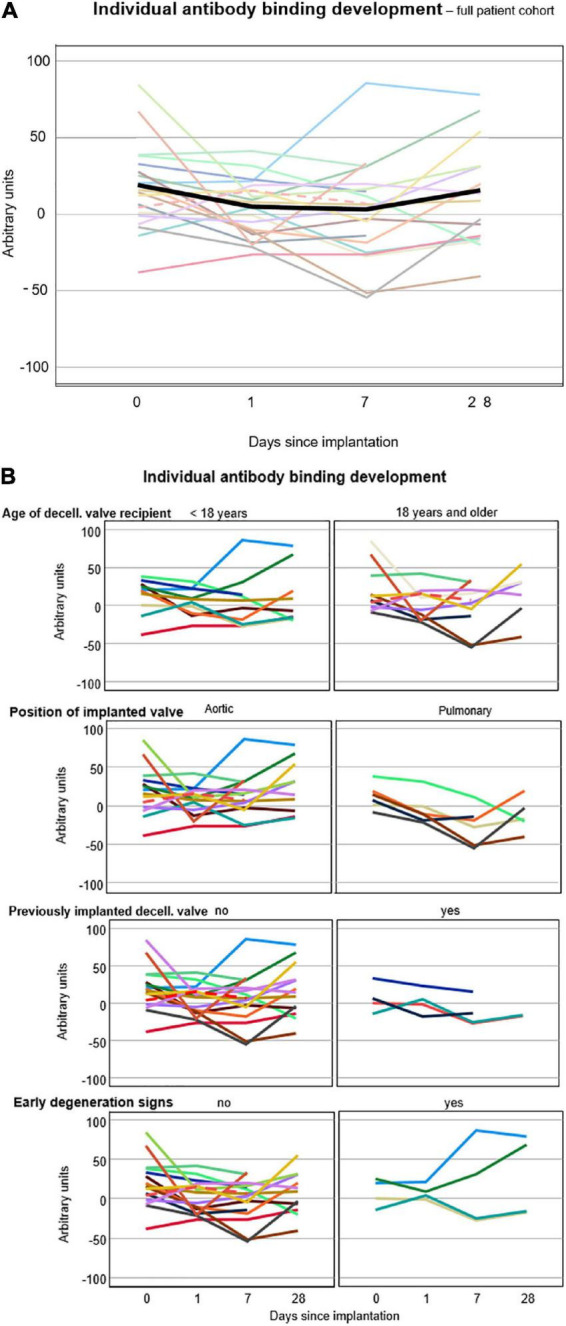
**(A)** Individual antibody binding of each patient against 12 solubilized decellularized valves. The lines connect the averages of all triplicate medians, displayed as arbitrary units, of each patient. The black line shows the average antibody binding of the whole study cohort at the respective time points. **(B)** Individual antibody binding for all 20 patients differentiated by age, valve position, prior DHV implantation, and echocardiographic signs of early degeneration. Broken lines indicate the patient post-lung transplantation.

Figures 3A,B exhibit the development of individual antibody binding for the overall study cohort, differentiated by age, valve position, prior DHV implantation, and echocardiographic signs of early degeneration.

Four patients, 3 following DAH implantation and 1 following DPH implantation, developed echocardiographic signs of early degeneration, such as cusp thickening, enhanced echogenicity, and/or mild flow acceleration during follow-up, which was 16.0 ± 4.7 months for the whole study group. All 4 patients were under 18 years of age (13.8 ± 1.6 years.), two of whom had received a decellularized allograft in the past (1 DAH, 1 DPH).

The antibody binding (median values of all triplets, in arbitrary units) did not differ significantly between recipients under and over 18 years: the median value under 18 years was 7.9 (IQR-12.2 to 29.0) and over 18 years was 5.3 (IQR-12.8 to 22.6); *p* = 0.602, Mann–Whitney *U*-test.

**FIGURE 4:**
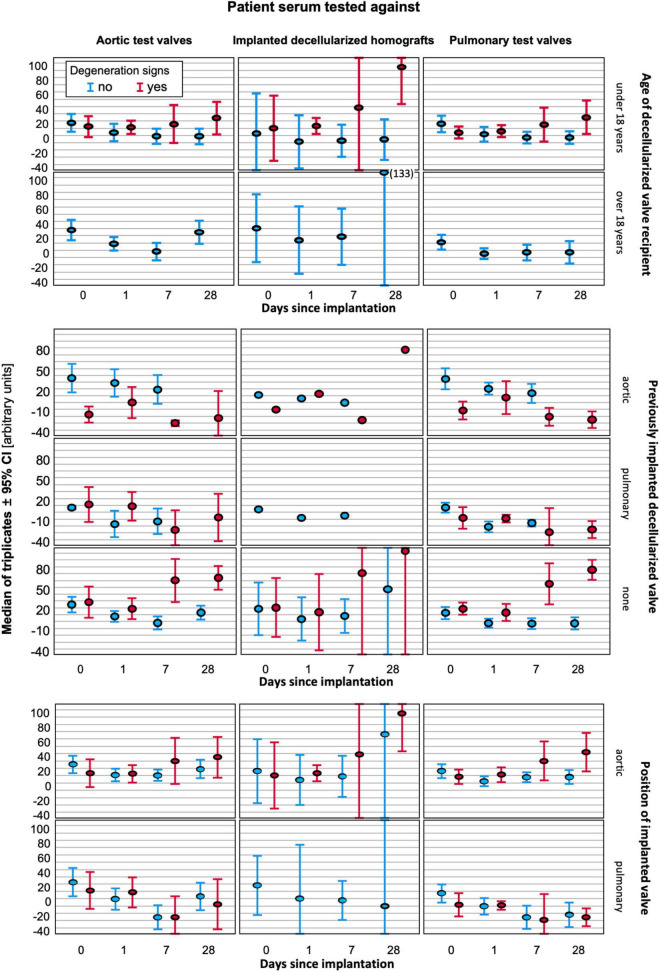
Antibody binding to implanted DHV (middle column) and test valves (left column-aortic, right column pulmonary) differentiated by age (superior block), valve position (middle block), and prior DHV implantation (inferior block). In each graph, the results for patients with signs of early degeneration are marked in red, and blue indicates no degeneration at the last follow-up.

Figure 4 provides a graphical comparison of the amount of antibody binding to the DHV implanted in the patient and graphical illustrations of the 5 non-implanted decellularized aortic and 6 non-implanted pulmonary test valves. The middle column shows the antibody binding to the implanted DHV, the left column displays the antibody binding to aortic test valves, and the right column displays the antibody binding to pulmonary test valves. Patients who exhibited early signs of degeneration are shown in red, and patients without degeneration are shown in blue. Results are also differentiated by age of the patients, prior DHV implantation, and valve position as outlined on the right side of the figure. A key finding is an increase in antibody binding in younger patients receiving decellularized aortic allografts. This increase was enhanced in patients with early degeneration signs, but this effect was not specific to the implanted DHV nor linked with previous DHV implantation.

In a stepwise logistic regression, attached as Supplementary Material, aortic position (odds ratio 3.216) and a previously implanted DHV (odds ratio 12.326) were the only significant factors relevant for the occurrence of early degeneration.

### Antibody binding toward explanted degenerated decellularized homograft valves samples and decellularized homograft valves retention samples prior to implantation

**FIGURE 5:**
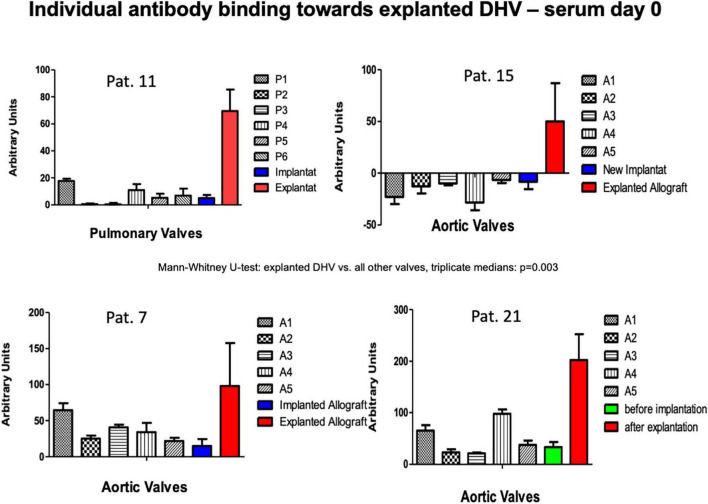
Antibody binding in patients with previous DHV and with new decellularized allografts replacing the degenerated previously implanted grafts (patients 7, 11, 15). The amount of antibody binding to one DAH retention sample 60 months after implantation and in the explanted degenerated DAH is also shown (patient 21).

In four patients, antibody binding to an explanted degenerated decellularized allograft was analyzed. In three of these patients, the amount of antibody binding to the explant was compared with the amount of binding to the newly implanted DHV. In patient no. 21, who had previously undergone a double semilunar valve replacement, a mechanical aortic valve replacement was performed due to DHV degeneration after 60 months. In this patient, who did not volunteer for the serial follow-up study, we were able to compare the results from the explanted aortic allograft with a retained decellularized sample prior to implantation. Figure 5 shows significantly increased antibody binding to the explanted DHV and lower binding to the non-implanted sample, indicating an antibody-mediated DHV degeneration.

### Test reproducibility

All tests were performed by one experimenter (FO). Technical triplicates were generated for all Dot-Blots. Experimental variance, as assessed by Cronbach’s α for Tau-equivalent reliability, was 0.979, indicating technical consistency. The interquartile range of the difference to the triplicate mean was −13.3 to 13.8 arbitrary units.

## Discussion

Decellularized allogeneic heart valves have shown markedly less antigenicity than cryopreserved allogeneic heart valves, e.g., no detectable donor-specific HLA antibody response, measured by Luminex-based single beads assay, was observed after implantation of decellularized allografts (10, 11). Decellularized allografts nevertheless appear to elicit a low-grade immune response as evidenced by the diminishing freedom from explantation after 10 years among patients in the ESPOIR Registry for decellularized pulmonary homografts (12, 13).

In healthy controls, we were able to show that preformed antibodies binding to decellularized human heart valves (DHV) are present even without previous exposure to allogenic material with high variability between individuals (7). In the current serial assessment study of antibody binding in patients undergoing heart valve replacement with DHV, we did not observe any influence in terms of donor age on the amount of patient-specific antibody binding to several different decellularized donor tissues. Regarding ABO blood type, we saw a higher amount of binding in recipients with blood group O, which, however, may be biased by the over-proportional prevalence of this blood group among the included patients.

The analysis of sex-specific factors for higher immunogenicity of DHV in male patients was limited in the current study as there were only 3 female patients among the 20 study participants. The finding of reduced antibody binding in patients with sex mismatch to the donor was unexpected and needs further analysis, in particular in the light of increasing recognition of sexual dimorphism in the immune system (12). In our study of preformed antibody binding in healthy controls, we also examined the impact of sex mismatch but did not find any clear links (unpublished data). In the serum of female control subjects, there was lower antibody binding in mismatch situations, whereas in the serum of male control subjects, higher results in mismatch situations were observed.

There was no significantly increased antibody binding observed in the overall antibody binding pre-operatively in the four patients who had previously received a decellularized allograft. When we tested these four patients post-operatively for antibody binding to their explanted homografts, we saw markedly increased antibody binding to their explanted decellularized allografts (two aortic and two pulmonary), which we believe strongly implicates an antibody-mediated immune reaction as a co-factor in decellularized allograft degeneration.

One of these patients, a female patient who had undergone a double semilunar valve replacement with decellularized allografts and, subsequently, explantation of the degenerated aortic allograft, was also tested against a retained sample of the decellularized aortic allograft, which had been implanted 5 years earlier. Interestingly, there was only mild antibody binding toward the non-implanted test samples, but a very strong reaction toward the explanted and degenerated aortic allograft, which shows the *de novo* synthesis of antibodies directed toward the aortic allograft after implantation.

During a serial assessment of the individual antibody binding levels at the first month after implantation, we noticed an increase in particular in patients following the implantation of decellularized aortic allografts. This finding indicates early activation of the humoral immune system of patients receiving decellularized allografts and correlates with our findings of higher rates of midterm degeneration in decellularized aortic allografts compared with pulmonary allografts (2, 13).

The amount of antibody binding was not higher in patients with prior DHV explantation. There, however, was a significantly increased amount of antibody binding after 28 days in 4 patients who, during the 19.8 months (maximum) follow-up available to date, showed mild echocardiographic signs of degeneration, such as cusp thickening and increased echogenicity of vascular parts. All 4 patients were under 18 years of age (13.8 ± 1.6 years.), which highlights the importance of age with respect to early degeneration of biological heart valves. As two of these patients previously received a DHV, the previous implantation appears to be a risk factor for early degeneration during logistic regression.

Unfortunately, this increased antibody binding was not specific to the implanted allograft, and we were unable to demonstrate a correlation between the pre-operative antibody binding to implanted grafts and poorer early echocardiographic results in subsequent follow-up. As one of our leading goals was to enable and optimize pre-operative matching, this was a disappointing finding. The binding intensity of the currently tested antibody assays seems to indicate rather than provide a prediction of degeneration.

These results, however, are based on a small cohort of 20 patients with a limited follow-up so far. We are currently trying to establish a lateral flow test kit to replace the elaborate Dot-Blot for a clinical study of the antibody-mediated immune response following implantation of a decellularized allograft. We are also analyzing the activation of several chemokines and cytokines after the implantation of decellularized allografts to further characterize the individual immune response pathways that may ultimately lead to graft degeneration. Ideally, such analyses should also include T-cell-mediated response. Such information, however, may be impossible to obtain as serial biopsies in delicate structures such as heart valves cannot be justified ethically given the overall good performance of decellularized allografts. Despite positive findings in terms of good recipient cell repopulation (4), we postulate that, in the early post-operative period, when the graft surface has not yet been fully covered by endothelial cells, the relatively open matrix of the decellularized tissue also may be penetrable by humoral antibodies, which could bind to the matrix and subsequently induce a T-cell and B-cell response or the activation of macrophages.

We hypothesize that the so-called matrikines, specific peptides of the extracellular matrix which may become visible to the immune system after processing, are the leading target for the innate and acquired immune system (14). We do not believe that cell surface proteins or protein fragments are the specific targets of preformed and new antibodies, as we quantitatively assessed these types of proteins (e.g., ULBP-4, NT5E, Siglec-9, Nectin-2) before and after decellularization and demonstrated their absence after the process (unpublished data). Further Western-blot analysis of explanted, degenerated decellularized homografts may be an option to identify the major binding domains of preformed and newly synthesized antibodies. Once there is a better understanding of the molecular mechanisms behind the immunological activation occurring after implantation of decellularized human heart valves, the opportunity may arise to silence the respective antigens before implantation (11). As the field of xenotransplantation is rapidly evolving, new initiatives using decellularized xenogeneic heart valves will likely play a role in the future (15), possibly in combination with autologous stem cell recellularization using induced pluripotent stem cells (iPS) (16, 17). Our group already has started to use decellularized heart valves from genetically modified pigs in primate models for pulmonary valve replacement.

## Limitations and conclusion

A limiting factor for the study was the low number of patients who could be included in the study due to the nature of homografts as a rare resource. This factor also limited the number of decellularized non-implanted tissue samples in comparison with the individual antibody binding.

Direct comparison of the results of antibody binding with healthy controls has technical limitations resulting from the variance in these complex experiments with the utilized biological test materials, e.g., such as different batches of secondary human antibodies. Direct comparison with the healthy controls was limited by different age and sex distribution within both groups.

Another limitation is the lack of long-term correlations of the early immunological activation, as evidenced by increasing antibody binding, with long-term DHV durability. We anticipate further insights in this regard in the future based on the ongoing follow-up of study participants.

In conclusion, serial assessment of tissue-specific antibody binding after implantation of decellularized allografts revealed increased antibody binding within 4 weeks after surgery in some patients, who subsequently developed early signs of allograft degeneration. Studies with a larger sample size are needed to confirm the prognostic relevance of antibody increase for valve performance and durability. In addition, specific research efforts to identify the molecular agents triggering this type of antibody response are required to understand the underlying processes.

## Data availability statement

The raw data supporting the conclusions of this article will be made available by the authors, without undue reservation.

## Ethics statement

The studies involving human participants were reviewed and approved by the Hannover Medical School Ethics Committee EC No. 7871_BO_S_2018. Written informed consent to participate in this study was provided by the participants’ legal guardian/next of kin.

## Author contributions

FO: investigation, formal analysis, and writing—original draft. RR, TG, and JE: validation and writing—review and editing. CF, AHo, MA, and DmB: investigation and writing—review and editing. RJ: writing—review and editing. DiB: formal analysis and writing—review and editing. AHa: funding acquisition, resources, and writing—review and editing. AHi: supervision, resources, and writing—review and editing. SS: conceptualization, supervision, and writing—review and editing. All authors contributed to the article and approved the submitted version.

## Footnotes

1
www.corlife.eu

2
https://www.medcalc.org
